# Work immersion and perceived stress among clinical nurses: a latent profile analysis and moderated mediation analysis

**DOI:** 10.1186/s12912-023-01467-7

**Published:** 2023-10-02

**Authors:** Yuan Liao, Wanting Wei, Sujuan Fang, Lihua Wu, Jing Gao, Xinyu Wu, Lijun Huang, Chun Li, Yu Li

**Affiliations:** 1 Guangzhou University of Chinese Medicine, Guangzhou, Guangdong Province 510006 China; 2 Guangdong Provincial Hospital of Chinese Medicine, Guangzhou, Guangdong Province 510120 China

**Keywords:** Clinical nurse, Work immersion, Perceived stress, Perfectionism, Social connectedness, Latent profile analysis, Moderated mediation analysis

## Abstract

**Background:**

Exploration of the relationship between individual work immersion and perceived stress is critical for clinical nurses’ effective psychological interventions and human resource management, as well as to alleviate nursing staff shortages. In order to further dissect the influencing factors of perceived stress among nursing staff, our study introduces the concepts of perfectionism and social connectedness to analyze the specific pathways that influence perceived stress in terms of an individual’s intrinsic and external personality traits. This study provides relevant recommendations for the development of stress management measures for nursing staff.

**Methods:**

This was a cross-sectional survey. 993 registered clinical nurses were included from four hospitals in Guangzhou through a convenience sampling method. Clinical nurses’ work immersion, perceived stress, perfectionism, and social connectedness were investigated using questionnaires based on latent profile analysis. The relationships between variables were analyzed using t-tests, analysis of variance, Pearson’s correlation analysis, latent profile analysis, and moderated mediation analysis.

**Results:**

The results showed that (1) general influences on nurses’ perceived stress included only child, labor relationship, labor allowance, and family support; (2) nurses’ work immersion contained four subgroups: lowest (12.6%), medium-low (39.8%), medium-high (39.9%), and highest (7.7%); (3) positive and negative perfectionism played a mediating role between the association of work immersion and perceived stress; (4) social connectedness played a moderating role in the mediation model of perceived stress.

**Conclusions:**

Work immersion, perfectionism, and social connectedness have an important impact on clinical nurses’ perceived stress. Nursing managers or leaders should pay attention to the differences of individual work immersion status, adopt reasonable stress management strategies, accurately identify positive perfectionist groups and strengthen the relationship between groups, so as to ensure the quality of nursing care, and reduce nursing turnover and alleviate the problem of staff shortage.

## Background

The shortage of nurses is a major global issue [1]. With the outbreak of COVID-19, the situation has become even more serious and the willingness of nurses to leave their jobs has been increasing [2]. Clinical nurses are constantly exposed to the uncertainty and risk of infection, as well as burdensome nursing tasks and caring for critically ill patients [3, 4], coupled with physical discomfort caused by wearing protective tools [5]. Nurses’ perceived stress levels are increasing and their psychological defenses are under constant assault [6], making nurses vulnerable to low retention intentions and physical or mental health problems, such as burnout, fatigue, acute stress disorder, anxiety, and depression [7–11]. This situation has led to an increase in the number of nurses choosing to leave the profession, resulting in a worsening the shortage of nurses, especially during the Covid-19 epidemic [12]. Increasing investment in nursing staff and job security have become important issues [13]. Therefore, it is crucial to analyze the influencing factors of nurses’ perceived stress and provide valuable insights for targeted psychological interventions and human resource management.

Stress is a common problem in health care, and perceived stress is a subjective expression of occupational stress. Cohen [14] suggested that perceived stress, as a state of tension generated by an individual in response to external environmental threats, is based on a combination of objective stressors, subjective stress perceptions, and stress responses, and is mainly manifested by tension and uncontrolled behavior. The presence and development of perceived stress has become an important influence on nurses’ professional identity and an important predictor of an individual’s physical and mental health [15–17]. Previous research on perceived stress in clinical nurses has focused on extrinsic factors such as significant workload, high-risk occupational environments, complex interpersonal relationships, ethical conflicts, patient mortality outcomes, and restricted work environment [18–23], while neglecting the role of intrinsic personal traits. Hammen and Padula [24, 25] suggested that personality traits and work engagement status may alter individual stress levels. Among them, work immersion, as an intrinsic professional quality of an individual’s approach to work, has become an important factor for many researchers exploring measures to reduce nurses’ stress [26, 27]. In addition, perfectionism, a personality trait that is often associated with a tendency to evaluate oneself critically, is particularly important in predicting individual stress levels [28]. Therefore, it is necessary to explore the effects of work immersion and perfectionism on clinical nurses’ perceived stress.

Work immersion is derived from the concept of Flow Experience [29], a transient pleasure experienced by individuals while performing work, which consists of concentration, work enjoyment, and intrinsic work motivation [30]. Related studies has shown that work immersion is negatively related to perceived stress [31, 32]. Intrinsic work immersion experiences may directly influence individuals’ perceived stress levels at work [25], and individuals with high levels of work immersion tend to be achievement and acquisition oriented, seeking value and meaning in their work [33, 34]. When facing work tasks, due to high mental focus and strong intrinsic work motivation, these individuals could turn work challenges into work enjoyment, thus reducing burnout and frustration, improving work quality, and ultimately alleviating their high perceived stress state [35, 36]. At the same time, work immersion is contagious [37] and can be spread among colleagues, thus contributing to the creation of highly engaged teams, increasing organizational commitment and job satisfaction, alleviating stress-generated discomfort, and reducing willingness to leave [38, 39].

Furthermore, work immersion experiences play an important role in shaping the personality trait of perfectionism [40]. Perfectionism is a personality trait that aims for high standards, endeavors to perform tasks to the best of its capability, and is frequently associated with the propensity for self-evaluation in a critical manner. Individuals with work immersion experiences tend to be driven by a high level of focus and intrinsic motivation, which motivates them to strive for higher goals and achieve perfectionistic outcomes [41, 42]. Perfectionism has also been shown to be a significant predictor of perceived stress in individuals [28, 43]. In earlier research, perfectionism had been considered to be a negative and maladaptive personality trait [44]. As research progressed, perfectionism was redefined as adaptive or maladaptive, that is, positive or negative perfectionism [45, 46]. The former refers to a willingness to accept potential failures and the belief that one’s self-esteem will not be severely impacted by the pursuit of goals, while the latter refers to excessive self-criticism and feelings of inadequacy in striving for high achievement and high standards, leading to negative emotions or psychological problems. Previous studies have mostly analyzed perfectionism as a single dimension [43, 47] and have primarily explored the relationship between perfectionism and individual health and mental illness [47, 48]. However, the two-way relationship of perfectionism received less attention. The development of psychological problems related to perceived stress have been less frequently studied from a two-way perspective of perfectionism, and no studies have investigated the mediating effect of perfectionism in the relationship between work immersion and perceived stress.

Therefore, healthcare organizations can implement strategies aimed at reducing the levels of stress among clinical nurses, so as to improved nursing work environment and reduced turnover rate by exploring the relationship between work immersion, perfectionism and perceived stress.

Additionally, social connectedness, defined as an individual’s self-perception of closeness to others in their surroundings [49], plays an active role in reducing perceived stress [50, 51]. Based on Maslow’s Hierarchy of Needs theory, the need for love and belonging is a fundamental human need, which leads individuals to actively seek out social connection that can satisfy this need, such as families and public welfare organizations, in order to alleviate negative emotions and positively regulate their state under high stress [52, 53]. Therefore, exploring social connectedness can provide deeper insight into the process of reducing perceived stress.

**Fig. 1:**
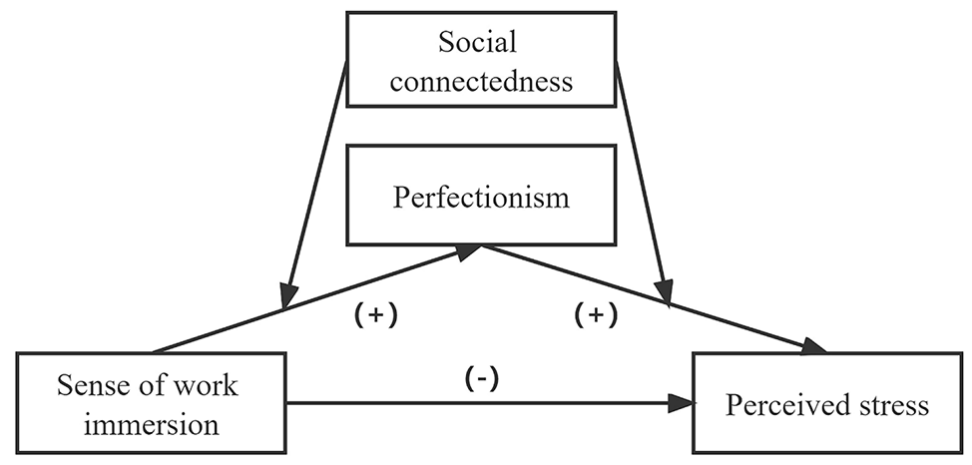
The conceptual model

In summary, the perceived stress of nursing staff continues to increase and the shortage of nursing staff is a major global problem. Work immersion, perfectionism, and social connectedness have been proven to be significant predictors of perceived stress. This study aims to explore the above four factors by incorporating them into a unified structural model and dissect the mediating effect of perfectionism and the moderating effect of social connectedness. This could fill the currently under-explained theoretical gaps, providing valuable insights for psychological interventions and human resource management for clinical nurses, as well as improving the nursing shortage problem. Based on the existing literature, the following hypotheses are proposed (Fig. 1):

### H1

There are significant associations between work immersion, perfectionism, social connectedness, and perceived stress.

### H2

The heterogeneity of work immersion can be identified through Latent Profile Analysis (LPA).

### H3

Perfectionism (positive and negative) mediates the relationship between work immersion and perceived stress.

### H4

Social connectedness plays a moderating role between work immersion and perfectionism (positive and negative) and perceived stress.

## Materials and methods

### Participants

A total of 1030 clinical nurses from four hospitals in Guangzhou were recruited between March and May 2023. 37 questionnaires were excluded due to non-response or incompleteness, resulting in a final sample of 993 (response rate 96.4%). Inclusion criteria were as follows: (1) those with hospital, contract or agency employment relationships; (2) those who have obtained a qualification certificate for clinical practice; (3) those who do not have cognitive dysfunction or psychiatric illness; and (4) those who have given informed consent and voluntarily participated in this study. Exclusion criteria: (1) nurse intern; (2) nurses undertaking training courses. The purpose of the study was explained verbally to obtain informed consent.

### Sample size

In this study, we adopted Yang’s conclusion that at least 50 subjects in each potential subgroup were needed to ensure accurate model fit information when conducting LPA or LCA [54]. Considering that there were 4 subgroups in this study, the required sample size should be at least 200, and an attrition rate of 20% should also be taken into account. Given the above conditions, the sample size of 993 subjects was valid for LPA-based analysis.

### Instruments

#### Demographic information

Based on previous literature [55–57], our study collected general demographic information (age, gender, marital status, etc.) and job-related information (labor relations, labor allowance, family support situation, etc.) from clinical nurses.

#### The work-related flow inventory (WOLF)

The WOLF was developed by Bakker [58], and the Chinese version was validated by Gu et al. (TLI = 0.96, CFI = 0.98, RMSEA = 0.05) [59]. The scale contains 13 entries and 3 dimensions: concentration (4 items, referring to an individual’s concentration at work), work enjoyment (4 items, referring to a person’s feelings of pleasure and positive perceptions at work), and intrinsic job motivation (5 items, referring to a person’s tendency to work for positive experiences or self-satisfaction) [60]. The scale is scored on a 5-point Likert scale ranging from 1 (not true at all) to 5 (almost always true), with the total score ranging from 13 to 65. The Cronbach’s alpha of WOLF in this study was 0.928, the Cronbach’s alphas for the dimensions ranged from 0.843 to 0.934.

#### The perceived stress scale (PSS)

The PSS developed by Cohen [61] is the most widely used tool for testing individual’s perceived stress, and the Chinese version is considered reliable [62, 63]. The scale contains 14 items and 2 dimensions: loss of control (all reverse items 4, 5, 6, 7, 9, 10, and 13) and sense of tension (items 1, 2, 3, 8, 11, 12, and 14). The scale is scored on a 5-point Likert scale ranging from 1 (never) to 5 (very often), and the total score ranges from 14 to 70, with higher scores indicating that the individual perceives more psychological stress. The Cronbach’s alpha for the PSS in this study was 0.827, and the two subscales were 0.915 and 0.881, respectively.

#### The frost multidimensional perfectionism scale (FMPS)

The FMPS was developed by Frost [44] and has 35 items. The Chinese version of the Frost Multidimensional Perfectionism Scale (CFMPS) had been translated and validated by Cheng et al. [64], and was later revised by Fei and Zhou [65]. The scale contains 27 items and 5 dimensions, including Concern for Mistakes (CM), Doubts About Action (DA), Personal Standards (PS), Parental Expectations (PE) and Organization (OR). CM, DA, PS, and PE constitute the negative perfectionism, OR constitutes the positive perfectionism [66]. It is a 5-point Likert scale ranging from 1 to 5, with a higher total score indicating a higher level of perfectionism. The Cronbach’s alpha of CFMPS in this study was 0.923, and the Cronbach’s alpha for each dimension ranged from 0.799 to 0.924.

#### The social connectedness scale (SCS)

The SCS was developed by Lee [67, 68]. A revised version (SCS-R) was developed in 2001, which contains 20 items and 2 subscales: social non-connectedness (all reverse items 1, 2, 3, 4, 5, 6, 8, 10, 15, and 18) and social connectedness (7, 9, 11, 12, 13, 14, 16, 17, 19, and 20). It is a 6-point Likert scale ranging from 1 to 6, with higher scores indicating higher levels of social connectedness. The SCS-R has been found to be reliable (CFI = 0.967, TLI = 0.944, SRMR = 0.031, RMSEA = 0.070) [69, 70]. The Cronbach’s alpha of the SCS-R in this study was 0.921, and the two subscales were 0.937 and 0.928, respectively.

### Statistical analysis

First, descriptive analysis was used to describe the general demographic and occupational profiles. In addition, Pearson’s correlation analysis was used to determine the correlation between work immersion, perceived stress, perfectionism (positive and negative), and social connectedness. The issue of common method variance (CMV) was also verified using Harman’s one-factor model [71].

Second, latent subgroups of nurses’ work immersion were identified through a latent profile analysis. Step-by-step profiling was performed based on the 1–5 categories of LPA model. The fitness metrics for evaluating the fit of the profile model were as follows [72, 73]: smaller values of the Akaike Information Criterion (AIC), Bayesian Information Criterion (BIC) and sample-adjusted Bayesian Information Criterion (aBIC) indicated better fit, while an entropy value of more than 0.80 indicated a classification accuracy of more than 90%. Lo-Mendell-Rubin adjusted likelihood ratio test (Lo-Mendell-Rubin, LMR) and sample-based Bootstrap Likelihood Ratio Test (BLRT) were used to compare the fit differences between the k-1 and k-category models, and a significant p-value indicated that the k-category model was better than the k-1 category model.

Finally, the mediating role of perfectionism between LPA-based work immersion profiles (categorical variable) and perceived stress was first evaluated through the PROCESS macro (Model 4) of SPSS. Subsequently, perfectionism was included into the regressions between work immersion (continuous variable) and perceived stress, and the moderating effect of social connectedness was fully examined by the PROCESS macro (Model 58). The total, direct, and indirect effects of the model were evaluated, and the mediating effect was considered statistically significant if the 95% bootstrap confidence interval did not contain zero [74].

All statistical analysis in this study were conducted on SPSS (version 26.0), SPSS PROCESS (version 4.0) and Mplus (version 8.3) software.

## Results

### Sample characteristics

A total of 1030 clinical nurses were initially included in this survey. Of these, 37 were then excluded due to missing data. The sex ratio of males to females was 1:20.59 and the average age was 33.42 ± 7.77 years old. 63.3% of the participants were married and 80.2% held a bachelor degree. The average level of perceived stress was 39.83 ± 8.29. There were significant differences in the variables related to the level of perceived stress among nurses, including being an only child (P = 0.013), hospital labor relations (P = 0.028), receiving labor subsidies (P < 0.001), and receiving family support (P < 0.001). The details are shown in Table 1.

**Table 1:** Demographic and professional characteristic differences in scores of perceived stress (N = 993)

Characteristics	*N* (%)	Perceived stress mean (± *SD*)	*P* value
Gender			
Male	46	40.48 ± 7.10	0.586
Female	947	39.80 ± 8.35	
Age			
19–30	415	40.25 ± 8.43	0.370
31–40	409	39.64 ± 8.16	
41–50	145	39.50 ± 8.06	
51–60	24	37.67 ± 9.46	
Only children			
Yes	249	38.70 ± 8.37	0.013
No	744	40.20 ± 8.24	
Marital status			
Single	344	40.59 ± 8.60	0.091
Married	629	39.45 ± 8.05	
Divorced	20	38.40 ± 9.82	
Education level			
College degree	179	40.12 ± 8.58	0.314
Bachelor degree	796	39.83 ± 8.24	
Master degree or above	18	37.00 ± 7.65	
Labor relationship with the hospital			
Authorized strength	254	39.69 ± 7.96	0.028
Contract employee	622	40.23 ± 8.46	
Service Dispatching(Third Party)	117	38.01 ± 7.91	
Whether you receive subsidies after working			
Yes	712	39.17 ± 8.24	<0.001
No	281	41.48 ± 8.21	
Whether your family supports your clinical nursing work			
Yes	870	39.17 ± 8.12	<0.001
No	123	44.50 ± 8.03	

### Common method variance test

This study used a self-assessment questionnaire and may suffered from common method bias. Therefore, Harman one-way factor analysis was performed to determine the presence of common method bias. An exploratory factor analysis was then performed on all study variables, and factors were extracted using the principal component approach. The results showed that there were 12 factors with characteristic roots greater than one, and the first factor explained 21.81% of the variance (less than the critical value of 40%), indicating that there was no serious common method bias problem in this study.

### Latent profile analysis of work immersion

**Fig. 2:**
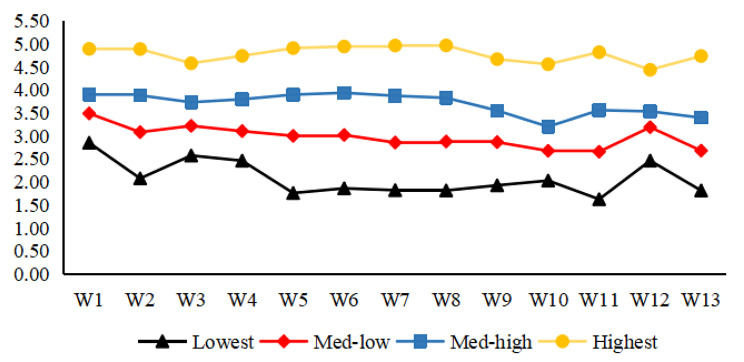
Probability of scoring on WOLF for 4 potential profiles of front-line nurses’ work immersion

As shown in Table 2, the LMR indicated that the four-profile model was a better fit than the three-profile model (P < 0.001). There was no significant difference between the four-profile model and subsequent models (P > 0.05), the entropy of the four-profile model was 0.944 and the value of BIC relatively small was relatively small, indicating accurate data classification. Therefore, the study identified the four-profile model as the best fit. The groups were named as lowest (12.6%), medium-low (39.8%), medium-high (39.9%), and highest (7.7%). Figure 2 shows the average scores of the four-profile model for each entry in nurses’ work immersion.

**Table 2:** Fitting index and group size of latent profile analysis models

Indices	Unconditional Model				
	1-profile	2-profile	3-profile	4-profile	5-profile
Fit statistics					
LL	-18533.613	-16478.316	-15717.900	-15020.528	-14743.403
AIC	37119.226	33036.631	31543.800	30177.056	29650.805
BIC	37246.645	33232.660	31808.439	30510.306	30052.665
aBIC	37164.068	33105.619	31636.933	30294.335	29792.230
Entropy	—	0.910	0.902	0.944	0.946
BLRT	—	0.0000	0.0000	0.0000	0.0000
LMR	—	0.0000	0.1036	0.0004	0.1561
Group-sizes(%)					
C1	993(100%)	486(48.9%)	148(14.9%)	125(12.6%)	77(7.7%)
C2	—	507(50.1%)	473(47.6%)	395(39.8%)	86(8.7%)
C3	—	—	372(37.5%)	396(39.9%)	354(35.7%)
C4	—	—	—	77(7.7%)	399(40.2%)
C5	—	—	—	—	77(7.7%)
C6	—	—	—	—	—

### Validation of the two-way perfectionism mediation model

**Fig. 3:**
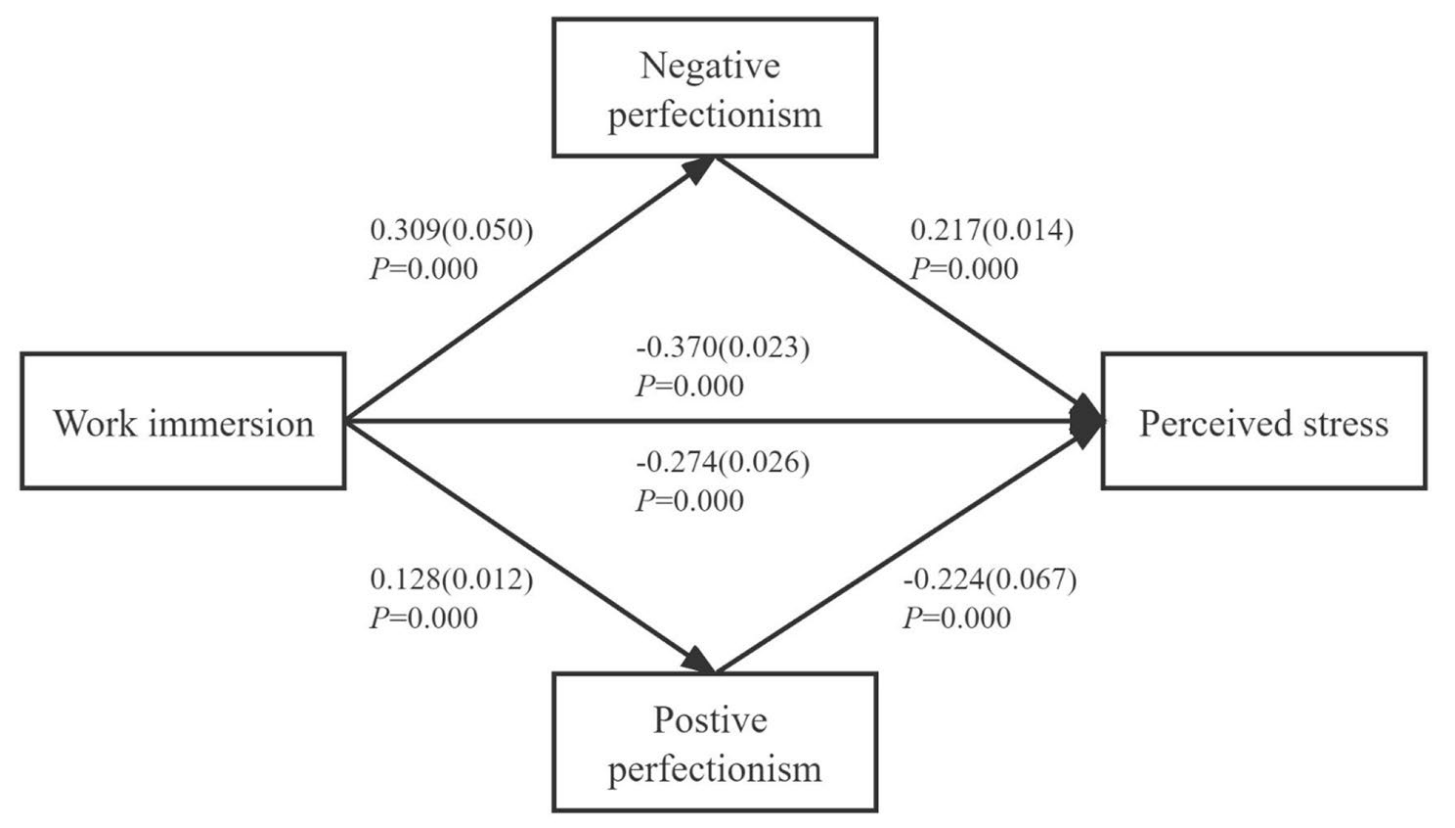
The mediating effect of perfectionism (including its two subscales) on perceived stress

We found a significant association between perceived stress, work immersion, positive perfectionism, and negative perfectionism (Table 3). This study was based on latent profile analysis and used the low subgroup of work immersion as the reference. The mediating effects of perfectionism were 0.138, 0.193, and 0.261 for the three subgroups, respectively. The 95% Bootstrap confidence intervals were (0.055, 0.234), (0.114, 0.281), and (0.125, 0.406), which did not contain “0”, demonstrating a significant mediating effect. Table 4 also shows the same results for both positive and negative perfectionism, further validating the perfectionism mediation model. The details are shown in Fig. 3.

**Table 3:** The level and association of nurses’ perceived stress with work immersion and perfectionism and social connectedness

Variables	Correlation Matrix							
	Mean	SD	1	2	3	4	5	6
1. Perceived stress	39.83	8.29	1					
2. Work immersion	42.77	9.73	-0.386 ^**^	1				
3. Social connectedness	86.69	17.47	-0.558 ^**^	0.290 ^**^	1			
4. Perfectionism	82.05	16.13	0.269 ^**^	0.242 ^**^	-0.357 ^**^	1		
5. Negative perfectionism	58.52	15.35	0.335 ^**^	0.172 ^**^	-0.469 ^**^	0.972 ^**^	1	
6. Positive perfectionism	23.53	3.81	-0.209 ^**^	0.332 ^**^	0.337 ^**^	0.318 ^**^	0.086 ^**^	1

Note. ^**^correlation is significant at the 0.01 level (2-tailed)

**Table 4:** The mediating effect of Perfectionism(categorical variable) on Perceived stress

Indirect effect	Effect (95%CI) 1 vs.2	Effect (95%CI) 1 vs.3	Effect (95%CI) 1 vs.4
LPM-Perfectionism-Perceived Stress	0.138 (0.055,0.234)	0.193 (0.114,0.281)	0.261 (0.125,0.406)
LPM-Negative Perfectionism-Perceived Stress	0.089 (0.004,0.182)	0.141 (0.062,0.227)	0.217 (0.076,0.364)
LPM-Positive Perfectionism-Perceived Stress	-0.057 (-0.108,-0.012)	-0.065 (-0.119,-0.014)	-0.065 (-0.125,-0.014)

Note. LPM = latent profile membership of work immersion

### Moderated mediation analysis of two-way perfectionism and social connectedness

**Fig. 4:**
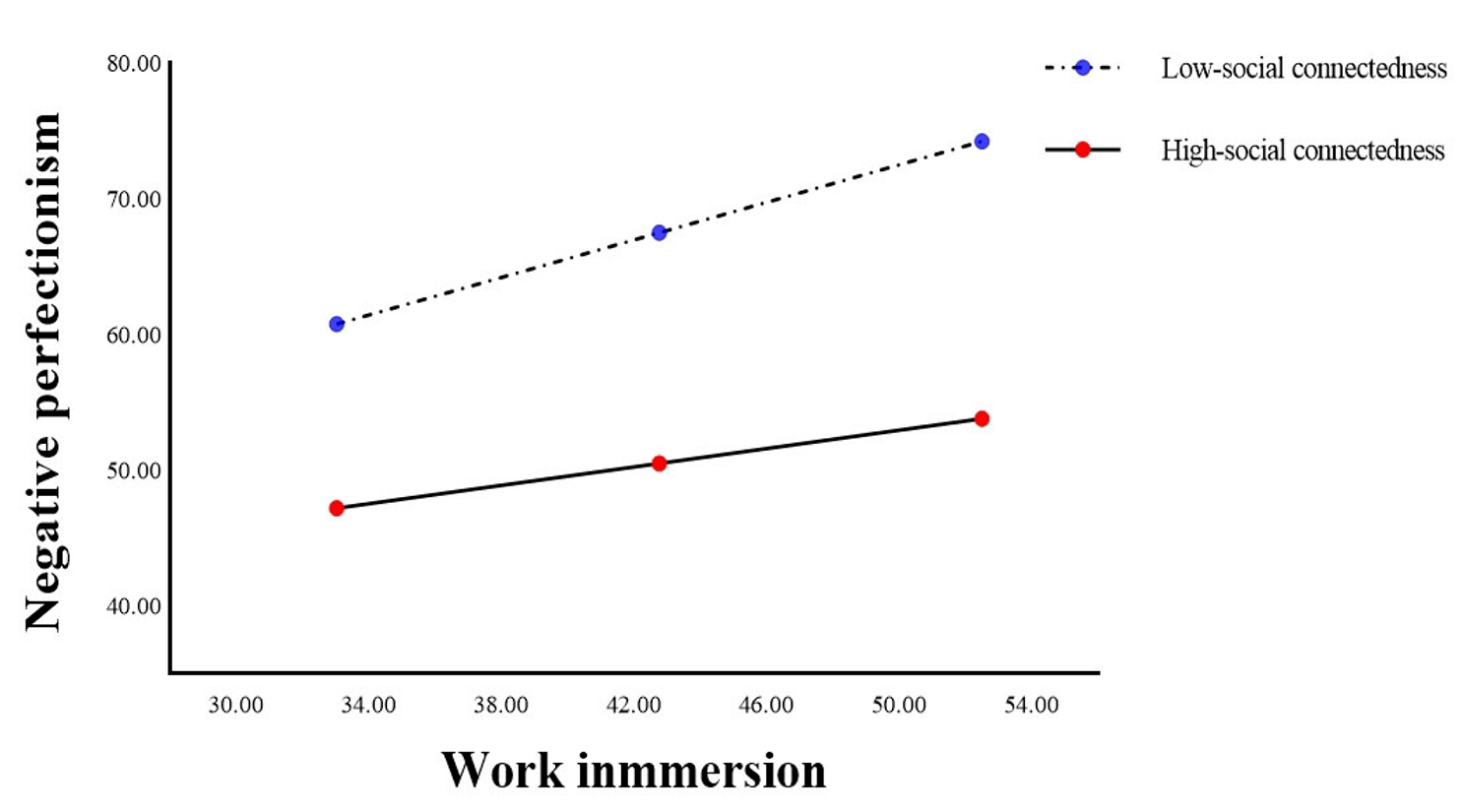
The interaction between work immersion and social connectedness on negative perfectionism

Table 5 showed that there was a significant interaction between work immersion and social connectedness (B=-0.010, SE = 0.002, 95% CI: -0.014, -0.006, P < 0.001), indicating that the relationship between work immersion and negative perfectionism was moderated by social connectedness (R^2^ = 0.163, F = 24.525, P < 0.001). The simple slope test (Fig. 4) revealed that nurses with high social connectedness (B = 0.341, SE = 0.059, t = 5.743, 95% CI: 0.224, 0.457, P < 0.001) had improved negative perfectionism when work immersion increased, compared to those with low social connectedness (B = 0.693, SE = 0.052, t = 13.242, 95% CI: 0.589, 0.794, P < 0.001).

**Table 5:** The moderated mediating effect of social connectedness through negative perfectionism on work immersion and perceived stress

Variables	Estimate	SE	*t*	*P*	LLCI	ULCI
	Moderating variable model (Outcome variable: Negative perfectionism)					
Constant	58.180	2.780	20.925	0.000	52.724	63.637
Work immersion	0.516	0.043	11.927	0.000	0.431	0.601
Social connectedness	-0.487	0.024	-20.228	0.000	-0.534	-0.440
Work immersion×Social connectedness	-0.010	0.002	-4.952	0.000	-0.014	-0.006
	Independent variable model (Outcome variables: Perceived stress)					
Constant	22.095	1.673	13.211	0.000	18.813	25.377
Work immersion	-0.370	0.023	-16.167	0.000	-0.415	-0.325
Negative perfectionism	0.217	0.014	15.096	0.000	0.189	0.245
Increase *R²* with interaction	*R²*		*F*		*P*	
	0.345		74.039		0.000	

**Fig. 5:**
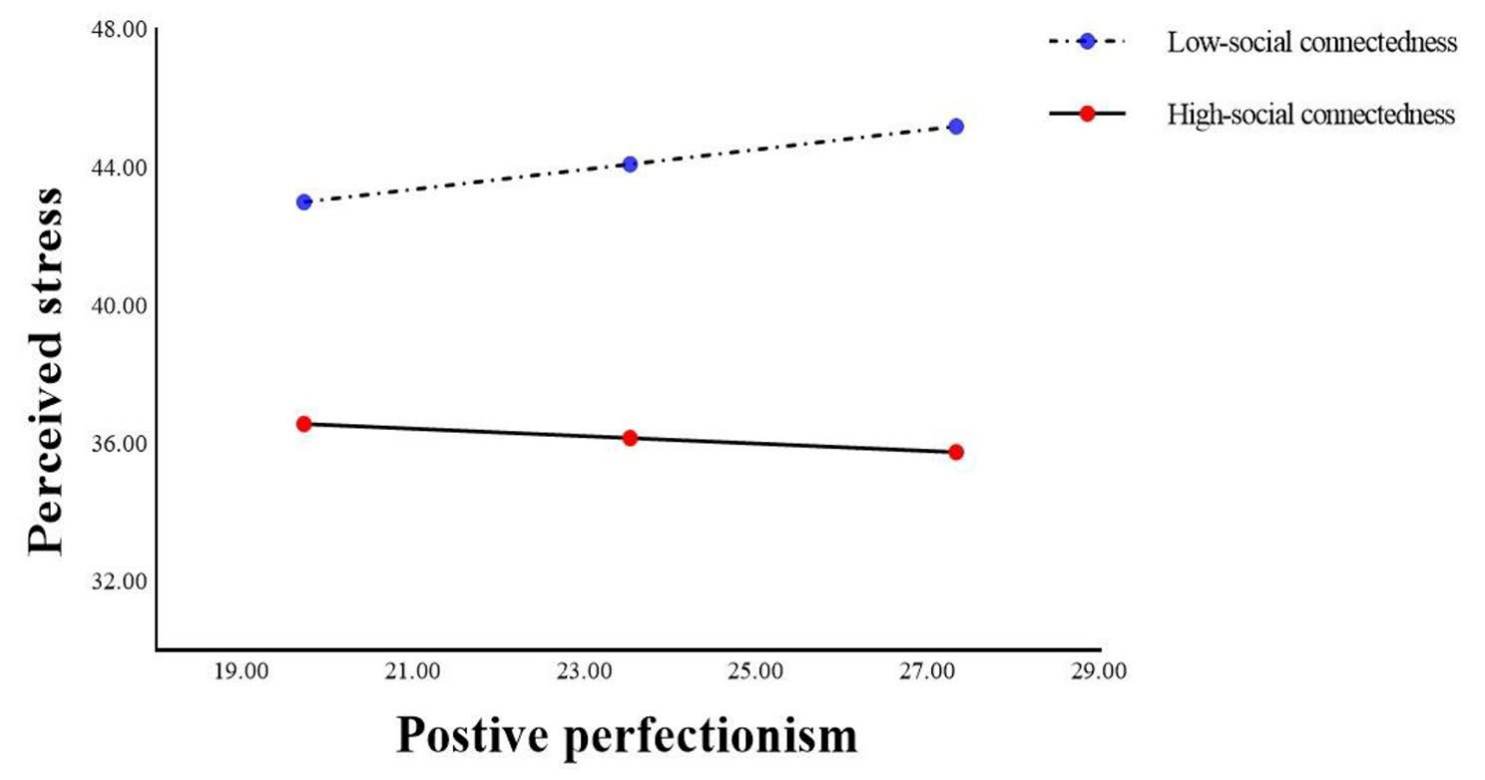
The interaction between positive perfectionism and social connectedness on perceived stress

Table 6 showed that there was a significant interaction between positive perfectionism and social connectedness (B=-0.011, SE = 0.003, 95% CI: -0.017, -0.005, P < 0.001), indicating that the relationship between positive perfectionism and perceived stress was moderated by social connectedness (R^2^ = 0.009, F = 13.925, P < 0.001). The simple slope test (Fig. 5) revealed that nurses with high social connectedness (B=-0.108, SE = 0.084, t=-1.292, 95% CI: -0.273, 0.056, P > 0.05) showed a decreasing trend in perceived stress as positive perfectionism increased, compared to those with low social connectedness (B = 0.290, SE = 0.078, t = 3.728, 95% CI: 0.137, 0.443, P < 0.001).

**Table 6:** The moderated mediating effect of social connectedness through positive perfectionism on work immersion and perceived stress

Variables	Estimate	SE	*t*	*P*	LLCI	ULCI
	Mediating variable model (Outcome variable: Positive perfectionism)					
Constant	-5.313	0.980	-5.422	0.000	-7.236	-3.390
Work immersion	0.128	0.012	10.881	0.000	0.105	0.151
	Dependent variable model (Outcome variables: Perceived stress)					
Constant	45.079	1.804	24.990	0.000	41.539	48.619
Work immersion	-0.209	0.023	-9.083	0.000	-0.254	-0.164
Positive perfectionism	0.091	0.061	1.495	0.135	-0.028	0.210
Social connectedness	-0.227	0.013	-17.187	0.000	-0.253	-0.201
Positive perfectionism×Social connectedness	-0.011	0.003	-3.732	0.000	-0.017	-0.005
Increase *R²* with interaction	*R²*		*F*		*P*	
	0.133		30.252		0.000	

## Discussion

In the current study, heterogeneity was observed in work immersion. With the inclusion of control variables, positive and negative perfectionism played a mediating role in the association between work immersion and perceived stress, while the two produced different mediating effects. Additionally, the paths that were mediated by social connectedness differed in the two types of mediation models. In other words, social connectedness may moderate the relationship between work immersion and negative perfectionism in the first type of mediation model, while in the second model, it may moderate the link between positive perfectionism and perceived stress.

Our study found significant associations between work immersion, perfectionism, social connectedness, and perceived stress in nurses, supporting the first hypothesis. Work immersion was negatively related to perceived stress, and this relationship has been confirmed in previous studies [31, 32].

This study also employed latent profile analysis to identify heterogeneity in work immersion of nurses and identified results for four latent subgroups, which is consistent with the findings of Yin et al. [75] and supported the second hypothesis. The results showed that more than 50% of nurses were in the low or moderately low work immersion group. Understanding the different work engagement levels of nurses could be useful for nursing managers to provide appropriate psychological interventions or support, such as emotional, material, and group support [76, 77].

The third hypothesis was confirmed by the mediating effect of positive and negative perfectionism. Our study showed that the relationship between work immersion (categorical variable) and perceived stress (continuous variable) was significantly mediated by perfectionism. Notably, negative perfectionism had a negative effect on the association between work immersion and perceived stress. An overly negative perfectionist personality undermines the positive effect of work immersion and exacerbates the perceived stress of nurses. This may be due to the fact that negative perfectionists tend to set goals that are too high or unreasonable, combined with the interaction of internal and external factors such as differences in individual resilience or high workloads, resulting in a highly stressful work experience [4, 16, 78]. Smith et al. [79] had also confirmed this finding in a meta-analysis of 11 longitudinal studies, noting that stress is an important condition for the elevated risk of anxiety in perfectionists. In contrast, positive perfectionists change negative emotions and perceived stress levels in a positive way [46, 80]. Therefore, it is necessary for nursing managers to differentiate between the types of perfectionism traits in nurses, rationalize work organization and human resource allocation, and avoid the occurrence and development of negative emotions in order to improve the quality of nursing work and reduce nurses’ stress levels [81]. This could be crucial to strengthen the willingness of nurses to stay in the workforce and alleviate nursing shortage [82].

The findings of this study were also consistent with the fourth hypothesis. Social connectedness has a moderating role in mediation models of both types of perfectionism. In the current study, for the mediation model of negative perfectionism, social connectedness moderated the association between work immersion and negative perfectionism, while indirectly alleviating nurses’ perceived stress. In contrast to previous findings, social connectedness did not directly alter nurses’ perceived stress levels [53, 68, 83]. Rather, social connectedness plays a direct positive role in influencing the main source of stress [84]. For the mediation model of positive perfectionism, social connectedness moderated the association between positive perfectionism and perceived stress, in other words, the interaction between social connectedness and positive perfectionism alleviated nurses’ perceived stress levels, which is consistent with the findings of Park et al. [51]. Therefore, dissecting the pathways in which social connectedness influences the alleviation of individual perceived stress based on the personality trait of perfectionism is an important way for nursing managers to provide effective psychological interventions.

### Limitations

This study has several limitations. First, the sample comprised only clinical registered nurses in China, which may differ from those in other countries. Hence, the results of this study cannot be generalized to other clinical registered nurses from diverse backgrounds. Second, the study design is cross-sectional, and therefore cannot establish causality between the variables. Further longitudinal or interventional studies are needed to confirm the associations found in this study. Third, in future related studies, the most suitable analytical approach for this type of research should be determined through the perspective of structural equation modelling. Lastly, the gender variables in the sample were unbalanced, which may pose a potential selection bias.

## Conclusion

Differences in perceived stress among clinical nurses are associated with several demographic factors (e.g., labor relations, labor subsidies, only child, and family support). Individual work immersion, social connectedness, and perfectionism traits have significant impacts on perceived stress among clinical nurses. Therefore, nursing administrators or managers should take a supporting perspective and provide appropriate financial support to nursing staff when needed, as well as facilitating communication and interaction between individuals and the outside world. Secondly, nursing decision makers should pay more attention to the dynamic changes of individual work immersion status from the perspective of individual differences, adopt appropriate stress management strategies, accurately identify positive or negative perfectionist groups, strengthen intergroup affinity, so as to ensure the quality of nursing work, reduce the turnover rate of nurses and alleviate the problem of staff shortage.

## Data Availability

The datasets generated and analyzed during the current study are not publicly available. The datasets are available from the corresponding author upon reasonable request.

## References

[1] World Health Organization (2020). State of the world’s nursing 2020: investing in education, jobs and leadership.

[2] Crowe S, Fuchsia Howard A, Vanderspank B (2022). The mental health impact of the COVID-19 pandemic on canadian critical care nurses. Intensive Crit Care Nurs.

[3] Nikeghbal K, Kouhnavard B, Shabani A, Zamanian Z (2021). Covid-19 Effects on the Mental workload and quality of Work Life in iranian nurses. Ann Glob Health.

[4] Wang X, Jiang X, Huang Q, Wang H, Gurarie D, Ndeffo-Mbah M (2020). Risk factors of SARS-CoV-2 infection in healthcare workers: a retrospective study of a nosocomial outbreak. Sleep Med X.

[5] Gao C, Ma G, Jiao D, Guo J, Zhang Y, Zhu L (2022). Investigation and analysis of occupational physical injuries among healthcare staffs during allopatric medical aid for the fight against COVID-19. Med Pr.

[6] Wu Y, Wang J, Luo C, Hu S, Lin X, Anderson AE (2020). A comparison of burnout frequency among Oncology Physicians and Nurses Working on the Frontline and Usual Wards during the COVID-19 epidemic in Wuhan, China. J Pain Symptom Manage.

[7] Molina-Mula J, Gonzalez-Trujillo A, Perello-Campaner C, Tortosa-Espinola S, Tera-Donoso J, De la Otero L (2022). The emotional impact of COVID-19 on spanish nurses and potential strategies to reduce it. Collegian.

[8] Holton S, Wynter K, Trueman M, Bruce S, Sweeney S, Crowe S et al. (2021). Psychological well-being of Australian hospital clinical staff during the COVID-19 pandemic. Aust Health Rev. 2021;45(3):297–305. 10.1071/AH20203. 10.1071/AH20203 33032681

[9] Murat M, Köse S, Savaşer S (2021). Determination of stress, depression and burnout levels of front-line nurses during the COVID-19 pandemic. Int J Ment Health Nurs.

[10] Salari N, Khazaie H, Hosseinian-Far A, Khaledi-Paveh B, Kazeminia M, Mohammadi M (2020). The prevalence of stress, anxiety and depression within front-line healthcare workers caring for COVID-19 patients: a systematic review and meta-regression. Hum Resour Health.

[11] Shahrour G, Dardas LA (2020). Acute stress disorder, coping self-efficacy and subsequent psychological distress among nurses amid COVID-19. J Nurs Manag.

[12] Yang J, Cheng Y, You Q, Liu C, Lai X, Zhang Y (2020). Psychological distress surveillance and related impact analysis of hospital staff during the COVID-19 epidemic in Chongqing, China. Compr Psychiatry.

[13] Daly J, Jackson D, Anders R, Davidson PM (2020). Who speaks for nursing? COVID-19 highlighting gaps in leadership. J Clin Nurs.

[14] Cohen S, Kamarck T, Mermelstein R (1983). A global measure of perceived stress. J Health Soc Behav.

[15] Jordan TR, Khubchandani J, Wiblishauser M (2016). The impact of perceived stress and coping adequacy on the Health of Nurses: a Pilot Investigation. Nurs Res Pract.

[16] Lara-Cabrera ML, Betancort M, Muñoz-Rubilar CA, Rodríguez Novo N, De las Cuevas C (2021). The Mediating Role of Resilience in the relationship between Perceived stress and Mental Health. Int J Environ Res Public Health.

[17] Zhu Y, Zhang Y, Wong FKY, Kuo SY, Cheung K, Lam MCH (2022). Newly graduated nurses’ stress, coping, professional identity and work locus of control: results of a cross-sectional study in Shanghai, Hong Kong and Taipei. J Nurs Manag.

[18] Al Sabei SD, Al-Rawajfah O, AbuAlRub R, Labrague LJ, Burney IA (2022). Nurses’ job burnout and its association with work environment, empowerment and psychological stress during COVID-19 pandemic. Int J Nurs Pract.

[19] González-Pando D, González-Nuevo C, González-Menéndez A, Alonso-Pérez F, Cuesta M (2022). The role of nurses’ professional values during the COVID-19 crisis. Nurs Ethics.

[20] Povedano-Jiménez M, Ropero-Padilla C, Rodriguez-Arrastia M, García-Caro MP (2021). Personal and emotional factors of nursing professionals related to coping with end-of-Life Care: a cross-sectional study. Int J Environ Res Public Health.

[21] Wu D, Jiang C, He C, Li C, Yang L, Yue Y (2020). Stressors of nurses in psychiatric hospitals during the COVID-19 outbreak. Psychiatry Res.

[22] Douglas MK, Meleis AI, Eribes C, Kim S (1996). The work of auxiliary nurses in Mexico: stressors, satisfiers and coping strategies. Int J Nurs Stud.

[23] Revicki DA, May HJ (1989). Organizational characteristics, occupational stress, and mental health in nurses. Behav Med.

[24] Hammen C (1991). Generation of stress in the course of unipolar depression. J Abnorm Psychol.

[25] Padula RS, Chiavegato LD, Cabral CM, Almeid T, Ortiz T, Carregaro RL (2012). Is occupational stress associated with work engagement ?. Work.

[26] Hetzel-Riggin MD, Swords BA, Tuang HL, Deck JM, Spurgeon NS (2020). Work Engagement and Resiliency Impact the relationship between nursing stress and burnout. Psychol Rep.

[27] Wang H, Xu G, Liang C, Li Z (2022). Coping with job stress for hospital nurses during the COVID-19 crisis: the joint roles of micro-breaks and psychological detachment. J Nurs Manag.

[28] Rice KG, Richardson CME (2014). Classification challenges in perfectionism. J Couns Psychol.

[29] Csikszentmihalyi M (1975). Beyond boredom and anxiety: experiencing Flow in Work and Play.

[30] Bakker AB (2005). Flow among music teachers and their students: the crossover of peak experiences. J Vocat Behav.

[31] Bernburg M, Hetzmann MS, Mojtahedzadeh N, Neumann FA, Augustin M, Harth V (2021). Stress perception, Sleep Quality and Work Engagement of German Outpatient Nurses during the COVID-19 pandemic. Int J Environ Res Public Health.

[32] Zhang M, Zhang P, Liu Y, Wang H, Hu K, Du M (2021). Influence of perceived stress and workload on work engagement in front-line nurses during COVID-19 pandemic. J Clin Nurs.

[33] Bargagliotti LA (2012). Work engagement in nursing: a concept analysis. J Adv Nurs.

[34] Hirschi A (2012). Callings and work engagement: moderated mediation model of work meaningfulness, occupational identity, and occupational self-efficacy. J Couns Psychol.

[35] Bonner L (2016). A survey of work engagement and psychological capital levels. Br J Nurs.

[36] Zhang N, Xu D, Li J, Xu Z (2022). Effects of role overload, work engagement and perceived organisational support on nurses’ job performance during the COVID-19 pandemic. J Nurs Manag.

[37] Bakker AB, Albrecht SL, Leiter MP (2011). Key questions regarding work engagement. Eur J Work Organ Psychol.

[38] Van Bogaert P, Wouters K, Willems R, Mondelaers M, Clarke S (2013). Work engagement supports nurse workforce stability and quality of care: nursing team-level analysis in psychiatric hospitals. J Psychiatr Ment Health Nurs.

[39] Van Bogaert P, van Heusden D, Timmermans O, Franck E (2014). Nurse work engagement impacts job outcome and nurse-assessed quality of care: model testing with nurse practice environment and nurse work characteristics as predictors. Front Psychol.

[40] Major DA, Turner JE, Fletcher TD (2006). Linking proactive personality and the big five to motivation to learn and development activity. J Appl Psychol.

[41] Bakker AB, Demerouti E (2007). The job Demands-Resources model: state of the art. J Manage Psychol.

[42] Demerouti E, Bakker AB, Nachreiner F, Schaufeli WB (2001). The job demands-resources model of burnout. J Appl Psychol.

[43] Wang Y, Chen J, Zhang X (2022). The relationship between perfectionism and social anxiety: a Moderated Mediation Model. Int J Environ Res Public Health.

[44] Frost RO, Marten P, Lahart C, Rosenblate R (1990). The dimensions of perfectionism. Cognit Ther Res.

[45] Dunkley DM, Blankstein KR, Zuroff DC, Lecce S, Hui D (2006). Self-critical and personal Standards factors of perfectionism located within the five-factor model of personality. Pers Individ Dif.

[46] Rice KG, Vergara DT, Aldea MA (2006). Cognitive-affective mediators of perfectionism and college student adjustment. Pers Individ Dif.

[47] Rasmussen KA, Slish ML, Wingate LR, Davidson CL, Grant DM (2012). Can perceived burdensomeness explain the relationship between suicide and perfectionism?. Suicide Life Threat Behav.

[48] Molnar DS, Sadava SW, Flett GL, Colautti J (2012). Perfectionism and health: a mediational analysis of the roles of stress, social support and health-related behaviours. Psychol Health.

[49] Abma TA, Baur V (2014). Seeking connections, creating movement: the power of altruistic action. Health Care Anal.

[50] Lee RM, Robbins SB (1998). The relationship between social connectedness and anxiety, self-esteem, and social identity. J Couns Psychol.

[51] Park D, Lee M, Osborne K, Minnick D (2023). Stress and depression in Ohio Social Workers during the COVID-19 pandemic: the buffering role of Social Connectedness. Health Soc Work.

[52] Humphrey A, March E, Lavender AP, Miller KJ, Alvarenga M, Mesagno C (2022). Buffering the fear of COVID-19: Social Connectedness mediates the relationship between fear of COVID-19 and psychological wellbeing. Behav Sci (Basel).

[53] Xiao Y, Zhang H, Li Q (2022). Role stress and psychological distress among chinese nurses during the COVID-19 pandemic: a Moderated Mediation Model of Social Support and Burnout. Front Psychiatry.

[54] Yang CC (2006). Evaluating latent class analysis models in qualitative phenotype identification. Comput Stat Data Anal.

[55] Huang ZP, Huang F, Liang Q, Liao FZ, Tang CZ, Luo ML (2023). Socioeconomic factors, perceived stress, and social support effect on neonatal nurse burnout in China: a cross-sectional study. BMC Nurs.

[56] Yang C, Yang L, Wu D (2023). The influence of grit on nurse job satisfaction: mediating effects of perceived stress and moderating effects of optimism. Front Psychol.

[57] Cheng P, Jasinski N, Zheng W, Wang L, Li L, Xu L (2023). Mental condition and function of resilience among families of frontline medical workers during COVID-19: potential influencing factors and mediating effect. J Affect Disord.

[58] Bakker AB (2008). The work-related flow inventory: construction and initial validation of the WOLF. J Vocat Behav.

[59] Gu H, Wen Z, Fan X (2020). Investigating the multidimensionality of the work-related Flow Inventory (WOLF): a bifactor exploratory structural equation modeling Framework. Front Psychol.

[60] Kang X, Yang L, Xu L, Yue Y, Ding M (2022). Latent classes of circadian type and presenteeism and work-related Flow differences among clinical nurses: a cross-sectional study. Psychiatry Investig.

[61] Cohen S, Kamarck T, Mermelstein R (1994). Perceived stress scale. Measuring stress: a guide for health and social scientists.

[62] Leung DY, Lam TH, Chan SS (2010). Three versions of perceived stress scale: validation in a sample of chinese cardiac patients who smoke. BMC Public Health.

[63] Long J, Liu TQ, Liao YH, Qi C, He HY, Chen SB (2016). Prevalence and correlates of problematic smartphone use in a large random sample of chinese undergraduates. BMC Psychiatry.

[64] Cheng SK, Chong GH, Wong CW (1999). Chinese Frost Multidimensional Perfectionism Scale: a validation and prediction of self-esteem and psychological distress. J Clin Psychol.

[65] Fei Z, Zhou X (2006). The chinese frost multidimensional perfectionism scale: an examination of its reliability and validity. Chin J Clin Psychol.

[66] Li J, Liu X, Yu B, Tang W, Liu X (2020). Attentional Bias for Imperfect Pictures in Perfectionism: An Eye-Movement Study. Front Psychol.

[67] Lee RM, Robbins SB (1995). Measuring belongingness: the social connectedness and the social assurance scales. J Couns Psychol.

[68] Lee RM, Draper M, Lee S (2001). Social connectedness, dysfunctional interpersonal behaviors, and psychological distress: testing a mediator model. J Couns Psychol.

[69] Sun X, Hall GCN, DeGarmo DS, Chain J, Fong MC (2021). A longitudinal investigation of discrimination and mental health in chinese international students: the role of social connectedness. J Cross Cult Psychol.

[70] Zhang Y, Dong K, Zhao G (2021). The mediating role of social connectedness in the effect of positive personality, alexithymia and emotional granularity on life satisfaction: analysis based on a structural equation model. Pers Individ Dif.

[71] Beasley TM (2014). Tests of mediation: paradoxical decline in statistical power as a function of Mediator Collinearity. J Exp Educ.

[72] Gitta L, Bengt OM (2007). Performance of factor mixture models as a function of model size, Covariate Effects, and class-specific parameters. Struct Equ Modeling.

[73] Williams GA, Kibowski F (2016). Latent Class Analysis and Latent Profile Analysis. Handbook of Methodological Approaches to Community-Based Research: qualitative, quantitative, and mixed methods.

[74] Hayes AF (2017). Introduction to Mediation, Moderation, and conditional process analysis: a regression-based Approach.

[75] Yin YZ, Lyu MM, Zuo M, Yao SY, Li H, Li J (2022). Subtypes of work engagement in frontline supporting nurses during COVID-19 pandemic: a latent profile analysis. J Adv Nurs.

[76] Cai Y, Li Q, Cao T, Wan Q (2023). Nurses’ work engagement: the influences of ambidextrous leadership, clinical nurse leadership and workload. J Adv Nurs.

[77] Giménez-Espert MDC, Prado-Gascó V, Soto-Rubio A, Psychosocial, Risks (2020). Work Engagement, and job satisfaction of nurses during COVID-19 pandemic. Front Public Health.

[78] Paul LH, Gordon LF, Kirk RB (1991). Perfectionism and neuroticism in psychiatric patients and college students. Pers Individ Dif.

[79] Smith MM, Vidovic V, Sherry SB, Stewart SH, Saklofske DH (2018). Are perfectionism dimensions risk factors for anxiety symptoms? A meta-analysis of 11 longitudinal studies. Anxiety Stress Coping.

[80] Stoeber J, Otto K (2006). Positive conceptions of perfectionism: approaches, evidence, challenges. Pers Soc Psychol Rev.

[81] Sawyer AT, McManus K, Bailey AK (2022). A mixed-methods pilot study of a psychoeducational group programme for nurse managers during the COVID-19 pandemic. J Nurs Manag.

[82] Oyeleye O, Hanson P, O’Connor N, Dunn D (2013). Relationship of workplace incivility, stress, and burnout on nurses’ turnover intentions and psychological empowerment. J Nurs Adm.

[83] Landmann H, Rohmann A (2022). Group-specific contact and sense of connectedness during the COVID-19 pandemic and its associations with psychological well-being, perceived stress, and work-life balance. J Community Appl Soc Psychol.

[84] Sarfraz M, Qun W, Sarwar A, Abdullah MI, Imran MK, Shafique I (2019). Mitigating effect of perceived organizational support on stress in the presence of workplace ostracism in the pakistani nursing sector. Psychol Res Behav Manag.

